# From Vulnerability to Hope: Experiences with COVID-19 over Time in Rural America

**DOI:** 10.3390/healthcare13212752

**Published:** 2025-10-30

**Authors:** Yodit Denu, Kathryn Moore, DenYelle Baete Kenyon, Susan E. Puumala, Chelsea Wesner, BreAnne A. Danzi

**Affiliations:** 1 Department of Psychology, University of South Dakota, Vermillion, SD 57069, USA; kathryn.moore2@va.gov (K.M.); breanne.danzi@usd.edu (B.A.D.); 2 Department of Public Health and Health Sciences, Center for Health Education, University of South Dakota, Vermillion, SD 57069, USA; denyelle.kenyon@usd.edu (D.B.K.); susan.puumala@usd.edu (S.E.P.); 3 Centers for American Indian and Alaska Native Health, Colorado School of Public Health, University of Colorado Anschutz, Aurora, CO 80045, USA; chelsea.wesner@cuanschutz.edu

**Keywords:** COVID-19, public health, rural community, mental health

## Abstract

**Background/Objectives:** The COVID-19 pandemic has impacted rural communities in unique ways. Those living in rural communities encounter several challenges in managing the effects of COVID-19, and exploring the perceptions of those from rural communities provides valuable information about rural health behaviors. This study aimed to explore the various reactions that individuals in a predominantly rural Midwestern state had regarding the COVID-19 pandemic and its impact on them at two time points (December 2020 and March 2021) during the initial rollout of the COVID-19 vaccination. **Methods:** Utilizing an inductive thematic approach to analyze data, researchers found several themes reflecting the participants’ reactions to COVID-19. **Results:** Participants described varying reactions to public health information around COVID-19 and how those reactions, and subsequent behaviors, were impacted by different rural values. The themes that emerged from the data were Vulnerability factors, Experiences of Emotions, Government Response, COVID-19 Guidelines, Politicization of Pandemic, and Hope/Optimism. **Conclusions:** The findings suggest the importance of a community-responsive approach to implementing public health interventions that align with community values and priorities. Using behaviorally based interventions that acknowledge individual experiences, beliefs, capacity/resources, and cultural norms may be effective in supporting promotive health behaviors in rural communities.

## 1. Introduction

Approximately 14% of United States residents live in rural counties (46.1 million people) [1], as defined by the U.S. Department of Agriculture [2] to be countryside, rural towns with a population of less than 2500, and urban areas with 2500 to 49,999 people not part of a larger metropolitan area. South Dakota is home to about 884,659 individuals, with about 10.7 individuals per square mile [3]. In 2020, it was estimated that 50.2% of South Dakota residents lived in rural areas [2]. When exploring barriers to health care and resources, there is much to consider. In South Dakota, 11.6% of the population lives in poverty, 12.2% of individuals under 65 are without health care, and 8.1% of individuals under 65 have a disability [2]. Health disparities exist across various rural environments due to problems such as poor infrastructure, limited resources and providers, limited transportation, lack of insured individuals, and a high poverty rate [3,4,5].

Coronavirus-19 (COVID-19) and its many variants have persisted in the United States since 2020 [6]. COVID-19 is an infectious disease transmitted through aerosol droplets, causing symptoms ranging from cold-like symptoms to severe respiratory illnesses. Since the start of the pandemic, COVID-19 has caused exponential deaths in the United States, affecting rural and urban areas differently [6,7]. Until the January 2022 surge of Omicron, reports indicated a steady increase in mortality rates in rural (nonmetropolitan) areas surpassing the death rates of metropolitan areas [8,9].

There have been many changes throughout the pandemic, including variations in news coverage, changes in regulations regarding masking and social distancing, and the creation and distribution of vaccinations [10,11]. While the news is essential in a rapidly changing pandemic, urban-centric pandemic news coverage may not be relevant to rural communities. It may make individuals in rural communities more likely to dismiss the virus and its impact on rural communities [12]. As these changes have occurred throughout the pandemic, it is essential to highlight the context of the time period that will be investigated in this study.

From the onset of the pandemic, South Dakota has taken a more hands-off public health approach. More emphasis was placed on promoting individual freedoms and allowing individuals to decide what works best for their families in terms of engagement with CDC-recommended health precautions [13,14,15]. The State of South Dakota did not initiate any state-wide “stay at home” order, mask mandate, or social distancing order. Instead, local governments were able to implement COVID-19-related orders independently. For example, during the summer of 2020, South Dakota was known for a “potential spreading” event at the Sturgis Motorcycle Rally [14].

Despite some local government efforts to prevent the spread of COVID-19 in rural communities, from December 2020 to March 2021, there was a drastic increase in mortality and incidence rates, even passing the mortality rates in urban areas [9]. However, the surge of COVID-19 cases in both urban and rural areas steadily decreased in the spring of 2021 [9]. At the same time vaccination rollout announcements began, with promises of vaccinations opening up for the general public in March of 2021 [10]. In South Dakota, there have been thousands of confirmed cases of COVID-19, with many individuals who died due to COVID-19 since the start of the pandemic. The vaccine rollout began in March 2021, and as of 24 March 2021, 19% (168, 245) of the population in South Dakota had received both doses of the vaccine, and 31% (274, 493) of the population received at least one dose [16].

Overall, COVID-19 has contributed to other sources of stress for individuals, financially, socially, physically, and psychologically [6,17,18]. There are several factors to consider when exploring behavioral health in rural areas, such as individual health behaviors, access to various services, environmental factors, and the surrounding context [19]. Such is also true when considering health behaviors related to other public health issues, such as the COVID-19 pandemic.

### COVID-19 Reactions

Individual reactions to pandemics vary depending on several contextual factors. These reactions are in response to life changes that have evolved since the pandemic’s start. Haleem et al. [20] separated these changes into three distinct categories: healthcare, economic, and social. An individual’s psychological well-being may be impacted across all categories. For example, the COVID-19 pandemic may increase the number of overworked medical professionals, significant financial losses, business closures, separation from support systems, and disruption of cultural and religious events [20]. All of these factors may impact one’s psychological well-being.

Additionally, COVID-19 has affected individuals differently depending on their development stage. At the pandemic’s beginning, the immediate concern was directed to individuals with health issues, with older adults placed in the category as most vulnerable. However, when viewing the pandemic from a life-course lens, it becomes evident that it is not only those with chronic health issues that are impacted by the pandemic. Individuals in all age groups have experienced disruption in their daily lives and, depending on their pandemic experience, may be more or less vulnerable to the disruption. Settersten et al. [21] highlight the areas they believe have the most impact on disrupting life trajectories and transition, including health, personal control and planning, social relationships and family, education, work and careers, and migration and mobility.

Despite some individuals’ resiliency to disruptions in routine and “normalcy,” many people experience adverse effects related to these disruptions, such as depression, anxiety, and PTSD [22]. Many of these reactions to the disruption of COVID-19 are expected, as people are enduring high-stress levels. A population-based survey of adults in the United States estimated that rates of depression symptoms have increased significantly from approximately 9% in 2018 to 28% in 2020 [23]. In September 2020, participants reported symptoms of anxiety or depression (33%), trauma or stress-related disorder symptoms (30%), increased substance use (15%), and suicidal intent (12%) [24]. Similarly, young adults (18–35) in the US from April to May 2020 reported high rates of loneliness (49%), depression symptoms (80%), anxiety symptoms (61%), and severe drug use (38%; [25]).

Qualitative literature exists exploring experiences of COVID-19 for rural older adults, parents and adolescents [26,27,28], specific COVID testing [29], and telehealth use [30,31]. Although minimal research has focused on reactions to COVID-19 among rural Americans, in a sample of 1009 respondents across the rural west, 53% of rural individuals did report some “negative” impacts on their overall life and 44% reported a negative impact on mental health, despite low rates of COVID-19 viral exposure in the sample [5]. However, little research has broadly focused on the lived experience of rural individuals during COVID-19. Given the unique challenges experienced in rural areas, this is an area where further research is needed.

This study was guided by the research question: what was the lived experience of rural individuals in the first year of the COVID-19 pandemic? The purpose of this study was to explore the various reactions that individuals in a predominantly rural Midwestern state (South Dakota) had regarding the COVID-19 pandemic during the initial rollout of the COVID-19 vaccination and how they have been impacted by the pandemic. In addition, this study sought to gain a foundational and preliminary understanding of factors that could impact rural communities’ response to COVID-19 and other public health issues, areas of potential barriers, and possible areas of culturally relevant interventions for the rural community.

## 2. Materials and Methods

### 2.1. Study Context

The participants from this current study came from a previous longitudinal study. The original study utilized a longitudinal survey design (at four time points) to collect data from adults living in South Dakota to explore the impact that the COVID-19 pandemic had on individuals in rural America. Participants were from 65 out of the 66 counties in South Dakota. Of South Dakota’s 66 counties, 30 are classified as rural and 34 are classified as frontier (less than 6 people per square mile). While the remaining 2 urban counties were not excluded from the study, South Dakota is a largely rural state [2,3]. The four time points when data were collected were March 2020, September 2020, December 2020, and March 2021. Data was collected using Qualtrics online survey software (2020/2021). No incentives were provided to participants. Participants had to be at least 18 years of age and currently residing in the state of South Dakota, which was verified by providing a home zip code within South Dakota. Individuals from outside of South Dakota were excluded from the study. Approximately 7000 individuals completed the survey, and 4761 responses were confirmed residents of South Dakota. See Danzi et al. [32] for more detail about the prior study.

### 2.2. Current Study

A subset of the original study was used for the current study. 440 of the 4761 participants responded to the open-ended question. Participant responses ranged from four words to 674 words. Participant responses with at least one sentence were eligible for analysis. A total of 207 participant responses to the open-ended question were analyzed for this study. A thematic analysis was conducted to identify what meaning participants made of their experience during the first year of the COVID-19 pandemic. Participants typed their responses, requiring no transcription procedure. The study pulled qualitative responses from the question, “Is there anything about your experience during the COVID-19 pandemic that you would like to share?” While not a traditional interview-based qualitative study, the authors hoped to develop an initial understanding of rural individual experiences. There are documented barriers when conducting research with rural individuals, including geographic isolation [33,34] social and cultural issues regarding privacy and anonymity for sensitive topics [19] and views of researchers as outsiders [34]. Online qualitative studies offer opportunities to reach participants who may typically be inaccessible due to mistrust, resources, and access [34]. Given documented barriers in rural communities and mistrust of outsiders, the open-ended response offers “felt anonymity” for those who may not want to discuss thoughts on COVID-19 face-to-face given its controversy [12,34,35,36].

The current study analyzed data collected from two of the four time points from the original study: December 2020 and March 2021. The two time points were chosen due to the timing of the initiation of the COVID-19 vaccine rollout in South Dakota and various opinions regarding the vaccination that the researchers hoped to observe in participant responses. This project was approved by the University of South Dakota Institutional Review Board, first as a public health surveillance project (IRB-20-111) then as a longitudinal study (IRB-20-125). Participants were provided with an informed consent form through the online survey software and clicked a button to indicate their consent. Participant names and signatures were not collected in order to protect the anonymity of participants.

Participants reported on their demographic characteristics, experiences with COVID-19, vaccination status and opinions, and mental health. Anxiety was assessed using the GAD-7 [37] and standard clinical cutoff scores were used. Respondents answer on a four-point Likert scale from 0 (Not at all) to 3 (Nearly Every day). Respondents are asked to report how often they have been bothered by listed problems in the last two weeks. Example items include “Feeling nervous, anxious, or on edge” and “Feeling afraid, as if something awful might happen” [37]. This scale showed satisfactory internal consistency (α = 0.92) [37]. Depression was assessed using the PHQ-9 [38] and standard clinical cutoff scores. Respondents answer on a four-point Likert scale from 0 (Not at all) to 3 (Nearly Every day). Respondents are asked to report how often they have been bothered by listed problems in the last two weeks. Example items include “Feeling down, depressed, or hopeless,” and “Feeling bad about yourself—or that you are a failure or have let yourself or your family down” [38]. This scale has adequate internal consistency (α = 0.89) [38].

### 2.3. Participants

Participants were pulled from a larger study exploring COVID-19 in South Dakota residents. The current study participants were eligible to participate if they were 18 years or older and a resident of the state of South Dakota and had completed the open-ended question. Participants were recruited via advertisements through several university listservs, large nonprofit organizations, healthcare organizations, social media, statewide public radio, government official endorsements, and the South Dakota Departments of Education and Health. Participants completed the study through the Qualtrics online survey software.

The current study sample (*n* = 207) was 75% female, and the mean age of participants was 54.14. The racial breakdown was as follows: 91% White, 1% American Indian and Alaska Native, 1% Pacific Islander, and 7% Other/Multiracial. Additionally, 30% of the sample were parents/caregivers of a child under age 18 and 27% were essential workers (i.e., workers in healthcare, law enforcement, public safety, food and agriculture, energy, water, and public transportation) [39]. There were no differences between the sample used for the qualitative analyses in the current study and the larger sample in gender (χ^2^ = 2.38, *p* = 0.304) or race (χ^2^ = 0.16, *p* = 0.689). However, the participants that were part of the qualitative analyses did tend to be older in age (*M* = 54.15, *SD* = 15.64) than the larger sample (*M* = 46.16, *SD* = 14.74), Welch’s *F* = 51.75, *p* < 0.001.

For both December 2020 and March 2021 respectively, there were no differences in anxiety between the qualitative sample (*M* = 4.90, *SD* = 4.61; *M* = 3.61, *SD* = 4.24) or the larger sample (*M* = 5.11, *SD* = 4.88; *M* = 4.19; *SD* = 4.99) at either time point (*F*[1, 566] = 0.23, *p* = 0.628; Welch’s *F*[1, 363.14] = 1.84, *p* = 0.176). For both December 2020 and March 2021 respectively, there were no differences in depression between the qualitative sample (*M* = 5.34, *SD* = 5.10; *M* = 4.25, *SD* = 4.71) and the larger sample (*M* = 5.46, *SD* = 5.27; *M* = 4.77, *SD* = 5.56) at either time point (*F*[1, 562] = 0.07, *p* = 0.798; Welch’s *F*[1, 359.66] = 1.17, *p* = 0.280). Table 1 depicts various characteristics reported by participants.

### 2.4. Data Management and Analytic Approach

The study utilized an inductive thematic approach to analyze data to propose a conceptual stress model for COVID-19. The authors utilized an inductive approach as the aim of this project was exploratory; there was no preexisting theory or concepts that the authors were hoping to examine. To explore the lived experience of rural individuals during COVID-19, the authors wanted participant narratives to emerge from the raw data. Additionally, using an inductive approach complements longitudinal qualitative research as it allows researchers to identify themes at a baseline and later to explore how and if experiences changed or stayed the same [40]. A constructivist epistemological lens guided this study as participant realties were explored through their written statements. The researchers did not assume the realities of participants; instead, participants detailed their experiences of COVID-19 and the various interpersonal, intrapersonal, and contextual factors that impacted their experiences.

While the coders/researchers did not have face-to-face interactions with participants, the coders were mindful of how their experiences may have influenced how data was coded and interpreted. Coder one is an African American woman, who is originally from a large city, while coder two is a White woman who comes from a rural background. Both coders at the time of this writing were completing their doctoral program in South Dakota, seeing patients and teaching students from the community. While the coders, to some degree had lived experience in a rural community, it is likely that other factors of the coders’ backgrounds influenced interpretation of data. Through frequent meetings and discussions in the coding and writing process, the coders supported each other in maintaining a reflexive approach.

Data were analyzed using Braun and Clarke’s [41] six-phase framework of thematic analysis: (1) Become familiar with the data by reading and rereading the transcripts and taking brief notes; (2) Generate initial codes to organize data in a meaningful way; (3) Find themes, which are significant patterns in the data; (4) Review and modify preliminary themes; (5) Finalize and define themes, exploring any relation to other themes and subthemes; (6) Produce and write up the report. Data collected in December 2020 and data collected in March 2021 were analyzed separately; December 2020 data were analyzed first, and upon completion of thematic analysis, March 2021 data was analyzed. Researchers manually coded the data. Two researchers independently coded a subset of the data and met to confirm the reliability of the coding process. Once reliability was confirmed, the two researchers coded the rest of the data and identified initial themes individually for both time points. Researchers discussed the themes, exploring and discussing any discrepancies until a consensus on themes was met. Researchers went through three iterations of data analysis until saturation was met.

## 3. Results

The study explored the experience of individuals living in South Dakota during the COVID-19 pandemic. Findings were collapsed into five themes for December 2020 data and six themes for March 2021 and are summarized in Table 2. Quotations were selected to highlight the findings of the thematic analysis. Pseudonyms have been assigned to maintain the anonymity of participants.

### 3.1. Theme 1: Vulnerability Factors

Participants identified numerous factors in their lives that impacted their experience during COVID-19. Individuals at both time points identified various life areas and factors that either contributed to COVID-19 stress or served as a protective factor. For example, one participant identified at the first timepoint (T1; December 2020), “The virus makes everything more stressful…My pay is being impacted. Just very tired and I just hope we can stay healthy until something changes” (“John,” 40m).

For others, the existence of factors such as job accommodations and financial stability, allowed them to feel “lucky” compared to others; one individual stated, “I am lucky in that I am retired, financially stable and comfortable with hobbies to keep me busy. COVID [sic] has so far been an inconvenience, not tragic for me. I know that could change very quickly” (T1, “Sarah,” 63f).

While only a few months passed between December 2020 and March 2021, many individuals reported they were still struggling at the latter time, identifying factors such as increased financial strain or contracting COVID-19. Others found the silver lining in identifying their “privilege” due to factors such as financial stability or community support. At Timepoint 2 (T2; March 2021) “Julia” (69f) described factors that influenced her experiences and emotions during the pandemic:

*Some of my anxiety has to do with things only tangentially related to COVID-19[sic]. e.g., my responsibilities in an organization changed because of the need to provide virtual rather than in person events. Family live very far away and travel limitations have made it impossible to visit. On a very positive note, some activities through church have been deeper and more fulfilling because of the virtual formal*.

While some participants identified that a stronger sense of community developed during the pandemic, other participants reported an increase in interpersonal conflicts due to disagreements about COVID-19.

### 3.2. Theme 2: Experience of Emotions

A common observation for participants at both timepoints was the emotional reactions individuals reported regarding their COVID-19 experience. Reactions tended to be either internalized emotional reactions or externalized reactions.

#### 3.2.1. Sub-Theme 1

Internalizing emotions. Participants highlighted the various emotions they felt inward, towards themselves, such as hopelessness and anxiety. One participant wrote, “It’s so hard knowing what is right to do as a parent. We are in person learning and having sports. My child is so worried about COVID [sic] but I don’t want [him] to be the only one in his class to go to online learning” (T1, “Jessica” 40f).

Individual experiences, such as parenting as “Jessica” described, and other responsibilities impacted how individuals experienced the COVID-19 pandemic. At timepoint two (March 2021), “Jane” (54f) shared what it had been like being an essential worker during this time:

*As an essential worker who works closely with an extremely vulnerable population, the past year has been very stressful. I have had great concern for my safety and the safety of our staff. We’ve all been exposed to COVID [sic], numerous times. At times, the stress is overwhelming*.

Participants described nervousness about spreading COVID-19 to loved ones, and others described worry about being exposed to COVID-19, like “Alan” (T1, 58m), who shared he experienced “Nightly dreams about being exposed to people in crowded rooms, restaurants, meetings, etc.”

Participants reported varying emotional reactions to the COVID-19 pandemic, and this was consistent across both time points, though more participants identified more externalizing emotions at the second timepoint.

#### 3.2.2. Sub-Theme 2

Externalizing emotions. For others, COVID-19 caused individuals to react emotionally to others like their family, friends, strangers, coworkers, individuals in the state and federal government, public health officials, and the media. Emotions reported include anger, disappointment, and embarrassment. A participant noted, “It is extremely frustrating and depressing. I am normally proud to live in South Dakota but I’m currently horrified and embarrassed at our behavior as a state” (T1, “Laura,” 47f).

While similar internalizing and externalizing reactions were present in December 2020 and March 2021, anger seemed to be reported more frequently in March 2021. In addition, participants highlighted the impact of such externalizing reactions, including being bullied by others:

*People that I thought I knew and were friends with (including a couple of family members) turned on me, insulted me and my intelligence, criticized me for refusal to adopt their conspiracy theories, anti-vaccination, COVID hoax theories. People in our church, with a few exceptions, treat us like 2nd class citizens for wearing masks, self-isolation etc.*

(T2, “Robert,” 74m)

One participant highlighted the changing emotional reactions individuals felt over time, from the start of the pandemic to March 2021:

*My reactions and feelings have changed over time. Early in the pandemic I developed anxiety issues. The anxiety has dissipated. I continue to feel angry at others in public because of the lack of precautions being taken…There are 22 dead from COVID in my world. I truly believe this could have been prevented. This is what angers me the most.*

(“Lydia,” 54f)

### 3.3. Theme 3: Government Response

Participants voiced their displeasure with the government and the role the government has had in handling the COVID-19 pandemic. There were two common but opposing reasons participants reported displeasure with their government officials: either the government was not doing enough and failed, as described by “Jackson” (T2, 39m):

*“South Dakota has handled COVID-19 worse than virtually any state. We probably have more than a thousand extra deaths because of the deplorable refusal to issue meaningful public health measures. The state engaged in dangerous anti-science policies, while trying to falsely portray themselves as doing a good job.”*

…or the government was too involved. Others noted their frustration at too much governmental influence, claiming there was “fear-mongering”. One individual wrote that there was “Too much fear mongering and attempts at overstepping governmental power” (T1,”Rebecca,” 68f). A minority of participants were pleased with the way the government handled the pandemic, citing they appreciated the opportunity to have “individual freedoms.” “Michael” (T1, 73m) shared “I am so glad our governor trusted the people to do the right thing. We are doing fine in SD, better off than most states.”

### 3.4. Theme 4: COVID-19 Guidelines

Many participants highlighted the effect health guidelines had on their COVID-19 experience, citing mistrust or misinformation and conflicting information regarding the guidelines. For example, individuals commonly cited a “lack of” education, public service announcements, literacy, or information. Many participants wrote of the conflicting information individuals were receiving. In December 2020, “Marie” (49f) shared that:

*There are so many conflicting professional recommendations that it’s getting confusing to know what guidelines we should be following. Recommendations for city areas vs. rural areas are completely different but yet we are being lumped together*.

“Marie” highlights that in addition to contradicting information being shared, she felt as if the some of the recommendations were not relevant to the rural community. The concerns regarding confusing or unclear information continued into the second time point. In March 2021, participants identified concerns about the vaccine rollout and its lack of information, “Janice” (T2, 70f) shared:

*I don’t know what to think. Between mass media and politics, they have confused this nation because no one knows what is true and no one knows how to to[sic] tell the truth to the people. We deserve better than mass hysteria. I have watched my friends suffer from anxiety issues for a year. All because of not getting straight answers about a vaccine that was hurriedly put together and not tested before putting it out to the public*.

### 3.5. Theme 5: Politicization of Pandemic

Many individuals reported politicizing the public health response (or lack thereof). Individuals cited governmental, state, and federal decisions and scientific programs as a cause and effect of this politicization. “Paul” (T2, 38m) reported, “The most stressful part of this pandemic has been the way that certain people in government and media have downplayed the virus and politicized public health responses.” “Simon” (T1, 55m) shared that it is a “…Sad day when someone uses this virus as a tool for political gain. I am republican and very disappointed.”

Some participants highlighted the necessity of health literacy for rural populations. “Kayla” (T2, 36f) stated:

*Just that a lack of science literacy and/or health literacy can be deadly for a society. As much as we blame political leadership (and we should continue to highlight those failings as well) we also need to keep in mind that the reason they were ‘believable’ in some of their misinformation was because many individuals did not have the media literacy or health literacy to locate and identify viable information. We can continue to improve upon this by incorporating it more into curriculum, but we need some sort of community program to reach the vast majority of adults over 18 that have not and will not be attending a university*.

While others highlighted concern with the politization of the pandemic, “Kayla” also emphasized factors that may influence the “politization” such as lack of health or science literacy. Importantly, she highlighted specific members of the community (i.e., adults over 18 who have not attended college) that may be underrepresented when health information is created and disseminated.

### 3.6. Theme 6: Hope

Many individuals reported they were hopeful in March 2021. For some individuals, they viewed the vaccine as a beacon of hope:

*I have been fully vaccinated for about 2 months now. Since being fully vaccinated, I have noticed that I feel a lot more comfortable, and my mental/physical health have improved since the peak of the pandemic… Just this week was the first time I didn’t feel anxious while being around someone outside of my circle who was not masked.*

(“Mandy” 29f)

This theme is consistent with the rate of individuals (75%) in the sample who reported they received the vaccine and those who had planned to get the vaccine (12%) as of March 2021 (see Table 1).

Some individuals highlighted the change they experienced over the pandemic, noting changes in their mental health and actions, such as socializing more due to increased comfort provided by the vaccine. “Maureen” (80f) noted she was:

*“Extremely anxious this time last year-followed our protocol of using hand sanitizer, daily temperatures, and masks. Limiting time in grocery stores, no social contact with family members. Since receiving both vaccines, and my COVID test Neg.[sic] I have a lot of my anxiety disappearing, feeling a lot more free.”*

## 4. Discussion

**Figure 1:**
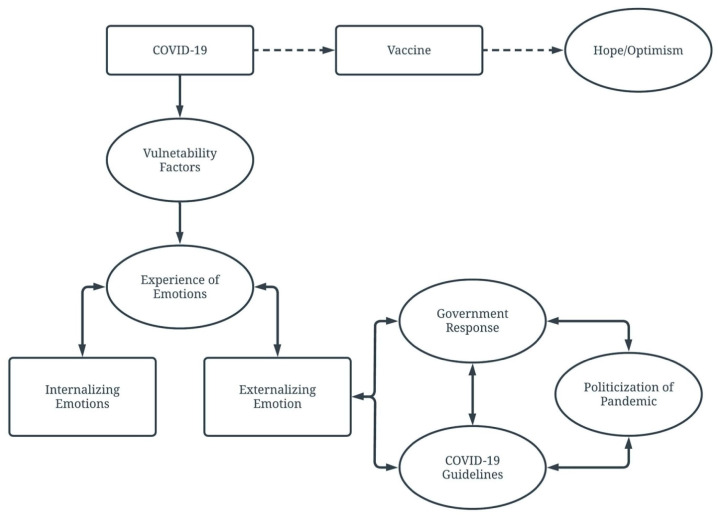
Conceptual Model of Qualitative Themes. Dotted lines indicate a theme only present in time point four (March 2021). The image depicts a conceptual model to demonstrate the relationship between the themes. Straight lines with arrows on both ends indicate a bidirectional relationship, curved lines with arrows on both ends signify it is related to the connected theme (i.e., internalizing emotions and externalizing emotions are connected to experiences of emotions.

To the best of our knowledge, this is one of the first projects to broadly explore the qualitative lived experience of rural residents in South Dakota during the first year of COVID-19. Results from this project suggest that for rural individuals, while the pandemic evolved, experiences and feelings evolved with it. This study highlighted the themes rural individuals consider important in their experience of the COVID-19 pandemic. The six themes were identified as Vulnerability Factors; Experiences of Emotions; Government Response; COVID-19 Guidelines; Politization of Pandemic; and Hope/Optimism. To aid in the interpretation of the findings, a conceptual model (Figure 1) was created that highlights the relationship between these themes. Many participants discussed themselves or their loved one’s experience with COVID-19; personal finances; their experience as caregivers to children, elderly parents, and disabled loved ones; and areas of social support (or lack thereof), representing the theme of Vulnerability Factors. As a result of these vulnerability factors, rural individuals appeared predisposed to different emotional reactions. Both internalizing and externalizing reactions were observed, falling under the theme of Emotional Reaction. This is consistent with what has been found in the literature. Specifically, those with less resilience are likely to suffer psychologically [22]. However, many people are more resilient to stress than others and will thus adapt and psychologically cope with the effects of a worldwide pandemic. Recent research demonstrates that individuals’ reactions to COVID-19 have varied significantly [17], and reactions to the pandemic have influenced the public’s adherence to policy, recommendations, and their ability to cope with distress (i.e., the threat of infection, transmission, losses).

While some reactions and comments stayed consistent from December 2020 to March 2021, there were notable changes in participants’ moods, behaviors, and feelings as they went into March 2021, almost one year since the recognition of COVID-19 in the United States. What was particularly interesting was that many participants identified externalizing reactions, especially towards others. For example, many reported being bullied or bullying others, turning their anger, frustration, and fear onto others or even leaders within the community. In addition, there was a bidirectional relationship between the Politicization of the Pandemic and COVID-19 Guidelines and Government Response. These factors seemed to influence the external reactions participants reported towards others in their community and government leaders and public health officials. Taylor [42] hypothesized that the psychological effects of a new pandemic would be more widespread than in past pandemics and speculated that individual reactions would fall on a spectrum, from those who disregard the impact of the disease and choose not to practice healthy behaviors to others’ experience of intense fear and possible debilitating distress. This is consistent with what was found in the current study. Participants shared varying reactions to the pandemic and associated regulations and public health interventions [42].

The communication of various health messages has its challenges such as sharing confusing and jargon-filled messages or communicating messages that have become politicized leading many consumers unmotivated to change or adjust their behavior [43,44]. Understanding consumer concerns, hesitations, and frustrations increase the likelihood of creating efficacious interventions. This is important to consider in areas or with populations that are perhaps underrepresented, such as rural communities where health literacy is lower compared to urban areas [45,46]. Health literacy is “the degree to which individuals have the capacity to obtain, process, and understand basic health information needed to make appropriate health decisions” [47]. The communication of health messaging has moved past just doctors sharing important health messages to the media, politicians, community members, and the internet. The challenge, for consumers, then becomes reviewing and identifying the appropriate and most accurate source.

As various political identities or values become involved in the discussion of health-related topics, it is likely the discussion will become polarized, representing differing political beliefs [43]. The politicization of health concerns and policies is not a new phenomenon. Debates have ensued on topics such as contraception, mammography screenings, human papillomavirus, and obesity [48,49,50,51].

The COVID-19 pandemic was influenced by political leadership opinions, media coverage, and partisan differences in their responses regarding nonpharmaceutical interventions [43]. Pharmaceutical responses were also politicized in the COVID-19 pandemic. The polarization of the pandemic itself was foundational in the politicization of the COVID-19 vaccine as well, influencing hesitancy and resistance to the vaccine [44]. As participants have highlighted, the messaging from their local, state, and federal guidelines have been inconsistent along with messages from various health professionals. Not only do these “confusing” guidelines and the politicized nature of the COVID-19 pandemic impact individual emotional reactions and their reactions towards others, but it impacts their understanding of what to do, when to do it, and who to trust.

### 4.1. Limitations

There are limitations of this study. The largest limitation is that the results are based on analysis of participant written responses to one question. While research has demonstrated online qualitative surveys as beneficial and an opportunity to increase access, there are limitations with analyses of responses from one question [19,35]. The qualitative responses were written by participants. This prevented the researchers from using a typical qualitative approach in which follow-up questions could be asked of the participants to further expand or elaborate on their experiences. This limited the depth of some responses. Moreover, completing an online survey with fill in the blank responses requires some level of literacy thus limiting those who may be able to respond [52]. Additionally, there was likely self-selection bias in the responses due to participants having the option to respond to a single open-ended question. Another limitation is that the data was analyzed roughly one year after data collection. Perceptions of COVID-19, vaccination, and other pandemic factors may have changed within that time.

While generalizability is not traditionally considered within the context of qualitative research [53,54,55], the authors would be remis to not consider how the interpretations would be different with other groups who live in rural communities. Firestone [56] and Polit and Beck [57] have identified strategies in qualitative research to generalize the process of human experiences as it relates to the study phenomenon. Groleau et al. [58] argues that narratives can supply a collective description around social experiences, including health, which can influence public health policies [59].

The conceptualization of how rural individuals experience COVID-19 may be limited as 91% of the participants identified as White. However, the study population is consistent with the racial makeup of South Dakota, according to the Census Bureau [3]. Age was also another factor that may impact representativeness as participants tended to skew older, which may have influenced their perceptions and experiences of the pandemic. The sample skewed toward women, representing 75% of the study sample, while the general population of South Dakota is 49.5% women [3], underrepresenting the experiences of men in South Dakota. These factors are important when considering how findings may be utilized to recognize how rural communities may make meaning of their health and factors that influence it.

### 4.2. Implications

When determining how to best implement an effective public health response, there are various cultural factors to consider in communicating the information and creating appropriate and relevant interventions for that specific community. There are major contributing factors to different infection and death rates in many rural areas. There are systemic and individual factors that contribute to the rate of deaths in rural areas, such as response efforts from state and federal governments, low vaccination rates, limited access to health care, lower rates of insurance, and higher poverty rates [5]. Individual factors include individual freedoms/decisions to not engage in preventive health behaviors (mask-wearing, social distancing), lack of trust in experts, and “charged partisan discourse” regarding COVID-19 [60]. Ideological differences in rural individuals may contribute to individual factors that impact COVID-19, such as personal values or political affiliation [60]. Values such as self-reliance, distrust of outsiders, religiosity, individualism, and fatalism are commonly found in rural areas [61,62]. While it is common for these values to be upheld by the majority of rural individuals, members of this rural community are not a monolith, such that they have other values that guide their behaviors and decisions. As found in the current study, there were mixed responses, and there were several participants who were concerned about the lack of public health responses in their community over their individual freedoms.

When thinking of the various values that are present in any community, it is important to not only think about the potential barriers that may arise when implementing public health interventions but also about the protective factors. One of the concerns that was brought up by participants was concerns regarding the vaccine and hesitation and refusal to get vaccinated. The World Health Organization office in Europe has identified a Tailoring Immunization Program (TIP) which utilizes a person-centered approach that attends to an individual’s experience and captures the complex, and at times idiosyncratic, nature of vaccine hesitation [63,64]. TIP is a behaviorally based intervention program that uses the Capability, Opportunity, Motivation, Behavior model (COM-B), which suggests that motivation (beliefs/emotions), capability (knowledge/skill), and opportunity (cultural norms/external factors) must be explored to influence healthy behavior [65]. TIP is used to identify at-risk groups that are hesitant to receive vaccines and the barriers that reduce vaccine uptake. Through this process, contextually, culturally, and evidence-informed interventions are implemented to increase vaccination uptake in at-risk communities. The TIP may be helpful in identifying the various idiosyncratic tendencies in the rural community and understanding why in March, while 67% were extremely comfortable with the vaccine, 75% had already received it and 12% planned to get the vaccine.

This study found there were varying emotional reactions, ranging from sadness, anger, disappointment, anxiety, and hopelessness, and beliefs regarding policies about COVID-19 that influenced (and were influenced by) the compliance with health behaviors. Rural individuals’ understanding of health policies, and who shares or recommends the health policies also impacted their motivation to engage in health behaviors and their actual health behaviors. And finally, the overall context of rural individuals and the various values and resources that were available to rural communities influenced engagement in motivation to engage in health behavior.

**Figure 2:**
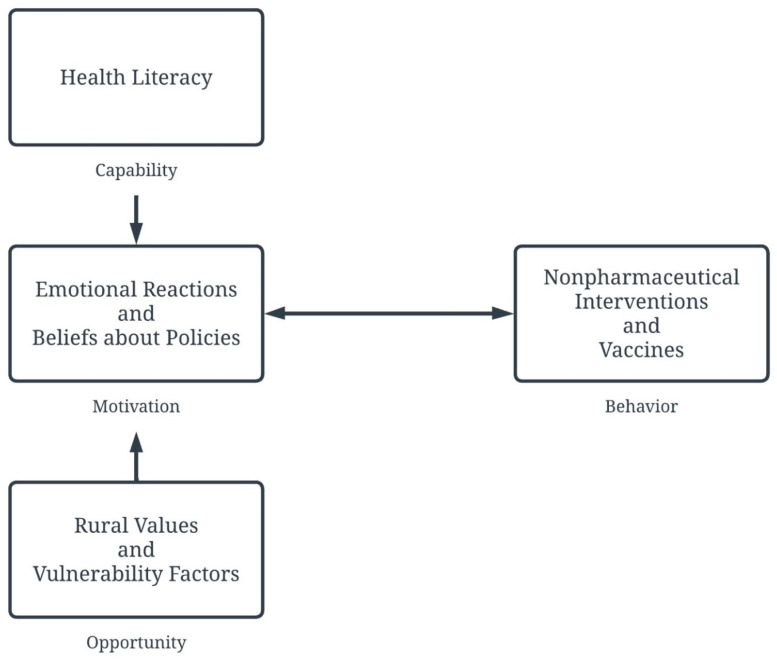
Conceptual Model of Behavioral Change using the COM-B Framework. This figure illustrates the COM-B framework of behavior change, utilizing themes found in the study as components that may change health behavior in rural communities.

Perhaps using a program similar to the Tailoring Immunization Program, which considers various cultural and idiosyncratic differences, can address some of the barriers to adherence to public health interventions. Acknowledging and exploring the rural motivation, emotions/beliefs, and opportunities may be beneficial in understanding engagement in both nonpharmaceutical interventions and pharmaceutical preventative health behavior. See Figure 2 for a conceptual model that may be useful to understand how to influence behavior change for rural individuals.

## 5. Conclusions

This study is among one of the first to examine the qualitative reactions that rural individuals had regarding the COVID-19 pandemic. Additionally, this study also offered a glimpse into how reactions changed over time, more specifically, how participants’ emotional reactions changed, both externalizing and internalizing, and the presence of hope and optimism following the distribution of the vaccine. Findings suggest the importance of a community-responsive approach to implementing public health interventions, such as psychoeducation or health communication strategies that align with community values and priorities.

## Figures and Tables

**Table 1:** Study Sample Characteristics.

	December 2020	March 2021
Psychological Symptoms		
Anxiety		
Minimal	53%	68%
Mild	35%	21%
Moderate	6%	8%
Severe	6%	3%
Depression		
Minimal	52%	62%
Mild	29%	27%
Moderate	12%	7%
Moderately Severe	6%	2%
Severe	1%	2%
COVID-19 Positive	11%	15%
With mild symptoms *	40%	36%
With moderate symptoms *	55%	56%
Hospitalized *	5%	8%
Comfort with Getting Vaccine Once Approved		
Extremely Uncomfortable	14%	10%
Somewhat Uncomfortable	16%	8%
Neutral	7%	4%
Somewhat Comfortable	23%	11%
Extremely Comfortable	40%	67%
Vaccination Status (Post-Approval)		
Received vaccination		75%
Planned to get vaccinated		12%
Did not plan to get vaccinated		13%

Note. Anxiety measured with the GAD-7 and Depression measured with the PHQ-9. * The statistics represents the percentage of those who had tested positive for COVID-19.

**Table 2:** Themes Identified from COVID-19 Experience in South Dakota.

Theme	December 2020 Codes	March 2021 Codes	Quote
Vulnerability Factors (+/−)	Poverty/financial strain; Caregiver stress; Essential worker; Pre-existing conditions; personal COVID-19 experience; isolation	Poverty/financial strain; Caregiver stress; Essential worker; Pre-existing conditions; personal COVID-19 experience; isolation; Interpersonal conflict; School Adjustment	“I am lucky in that I am retired, financially stable and comfortable with hobbies to keep me busy. COVID has so far been an inconvenience, not tragic for me.” (“Sarah,” 63f, December 2020)
Experience of Emotions	**Internalizing** (depression, lonely, helpless, hopeless, nervous); **Externalizing** (anger, fear, disappointment) Bullying	**Internalizing** (anxiety, confusion, lonely, stress, hopeless, shame); **Externalizing** (anger, frustration, concern, disappointment, embarrassed) Bullying	“My reactions and feelings have changed over time. Early in the pandemic I developed anxiety issues. The anxiety has dissipated. I continue to feel angry at others in public because of the lack of precautions being taken” (“Lydia,” 54f, March, 2021)
Government Response	Disgust with government, embarrassed by government/state of SD, disappointed by handling of pandemic, too much/not enough governmental influence	Frustrated/angry toward government, Resentful of lack of leadership, happy to not be affiliated with SD, too much/not enough governmental influence	“It’s a slap in the face when you work so hard to fight this disease, and your government leaders are not behind you” (“Gabi,” 35f, December, 2020)
COVID-19 Guidelines	Misinformation, distrust of science/CDC, conflicting COVID-19 recommendations	Misinformation, not enough information on vaccine	The worst part of COVID has been the misinformation and outright lying by some of the people and organizations I have believed in most of my life. “Scientific data” has been so muddled with untruths and half-truths as to be no better than fairy tales. The resultant anger and panic has led to my own anger and distrust of all “facts” (“Jeff,” 64m, March, 2021)
Politicization of Pandemic	Democratic lie, politicization of pandemic/COVID-19/virus, politicization makes people not adhere to safety	Distrust, lack of separation from health and politics, public health response politicized, need more public health policies, solution suggestions	“…the politics and drama related to the National election, polarization around party lines, the politicization of mask-wearing and vaccinations, and social justice is not helping to stay calm and to cope with an already stressful world.” (“Molly,” 68f, December, 2020)
Hope/Optimism	n/a	Relief, vaccination provided comfort, improvement in mental/physical health, hope about end of pandemic	“Since receiving both vaccines, and my COVID test Neg. I have a lot of my anxiety disappearing, feeling a lot more “free”” (“Maureen,” 80f, March, 2021)

Note. Bolded words indicate a sub-theme.

## Data Availability

The data presented in this study are available on request from the corresponding author due to privacy and ethical reasons, specifically that details provided in the qualitative responses could be potentially identifiable and that the consent form for the study did not include permission to make the data publicly accessible in a repository.
